# Selection and Characterization of SARS‐CoV‐2 Spike Binding Clickmers

**DOI:** 10.1002/cbic.202500733

**Published:** 2026-01-09

**Authors:** Nima Moradzadeh, Anna Jonczyk, Anton Schmitz, Volkmar Fieberg, Laia Civit, Julián Valero, Michael Famulok, Günter Mayer

**Affiliations:** ^1^ Life & Medical Sciences (LIMES) Institut Chemical Biology & Medicinal Chemistry Unit Universität Bonn Gerhard‐Domagk‐Str. 1 53121 Bonn Germany; ^2^ Interdisciplinary Nanoscience Center (iNANO) Aarhus University DK Aarhus 8000 Denmark; ^3^ Department of Molecular Biology and Genetics Aarhus University DK Aarhus 8000 Denmark; ^4^ Center of Aptamer Research & Development University of Bonn Gerhard‐Domagk‐Str. 1 53121 Bonn Germany

**Keywords:** aptamers, chemically modified aptamers, clickmers, Covid‐19, SARS‐CoV‐2 spike protein

## Abstract

Expanding the chemical repertoire of canonical nucleotides is key to unlocking the full functional potential of aptamers for diagnostic use. Herein, click‐systematic evolution of ligands by exponential enrichment (SELEX) is employed to generate chemically modified DNA aptamers, termed clickmers, that target the SARS‐CoV‐2 spike (CoV2‐S) glycoprotein. Two independent split‐combine selection strategies yield distinct clickmer families functionalized with benzofuran or indole moieties. Lead candidates (BF1 and N2) demonstrate nanomolar affinity for wild‐type CoV2‐S and maintain binding to multiple variants, including Alpha, Delta, and Mu, as validated by flow cytometry, surface plasmon resonance, and microscale thermophoresis. Structure–function analysis reveals essential click‐in positions for both full‐length clickmers and a truncated N2 variant, as short as 31 nucleotides, which displays increased binding to the Omicron variant. These results highlight the versatility of the click‐SELEX platform and exemplify its successful application to a clinically relevant target, advancing previous developments in the field.

## Graphical abstract

Split‐combine click‐systematic evolution of ligands by exponential enrichment against wild‐type SARS‐CoV‐2 spike glycoprotein yields benzofuran‐ and indole‐functionalized DNA aptamers (clickmers). These clickmers retain specific binding to multiple variants with nanomolar affinity, and the 31 nt indole‐functionalized truncate T2 retains binding to Omicron, highlighting the versatility of the platform for diagnostic aptamers.

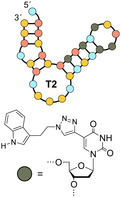

## 1 Introduction

The ongoing development of high‐affinity molecular recognition elements remains a cornerstone of biotechnology, diagnostics, and therapeutic design. Since the outbreak of the COVID‐19 pandemic, a wide range of nucleic acid aptamers have been reported targeting the SARS‐CoV‐2 spike (CoV2‐S) glycoprotein, including both DNA‐ and RNA‐based sequences.^[^ ^1^ ^,^ ^2^ ^]^ While many of them exhibit promising binding affinities and biological activity, their interaction properties are constrained by the limited chemical diversity of canonical nucleotides.

We identified chemical diversified aptamers termed clickmers^[^ ^3^ ^]^ to expand the chemical repertoire of spike‐binding oligonucleotides. These synthetic aptamers incorporate 5‐ethynyl‐2′‐deoxyuridine (EdU) in place of thymidine, providing a terminal alkyne handle for orthogonal modification via copper(I)‐catalyzed azide–alkyne cycloaddition (CuAAC). This strategy enables site‐selective conjugation of a wide range of functional moieties, referred to as “click‐ins”, directly into the nucleic acid backbone, significantly expanding the chemical and functional landscape accessible to aptamer scaffolds.

**Figure 1:**
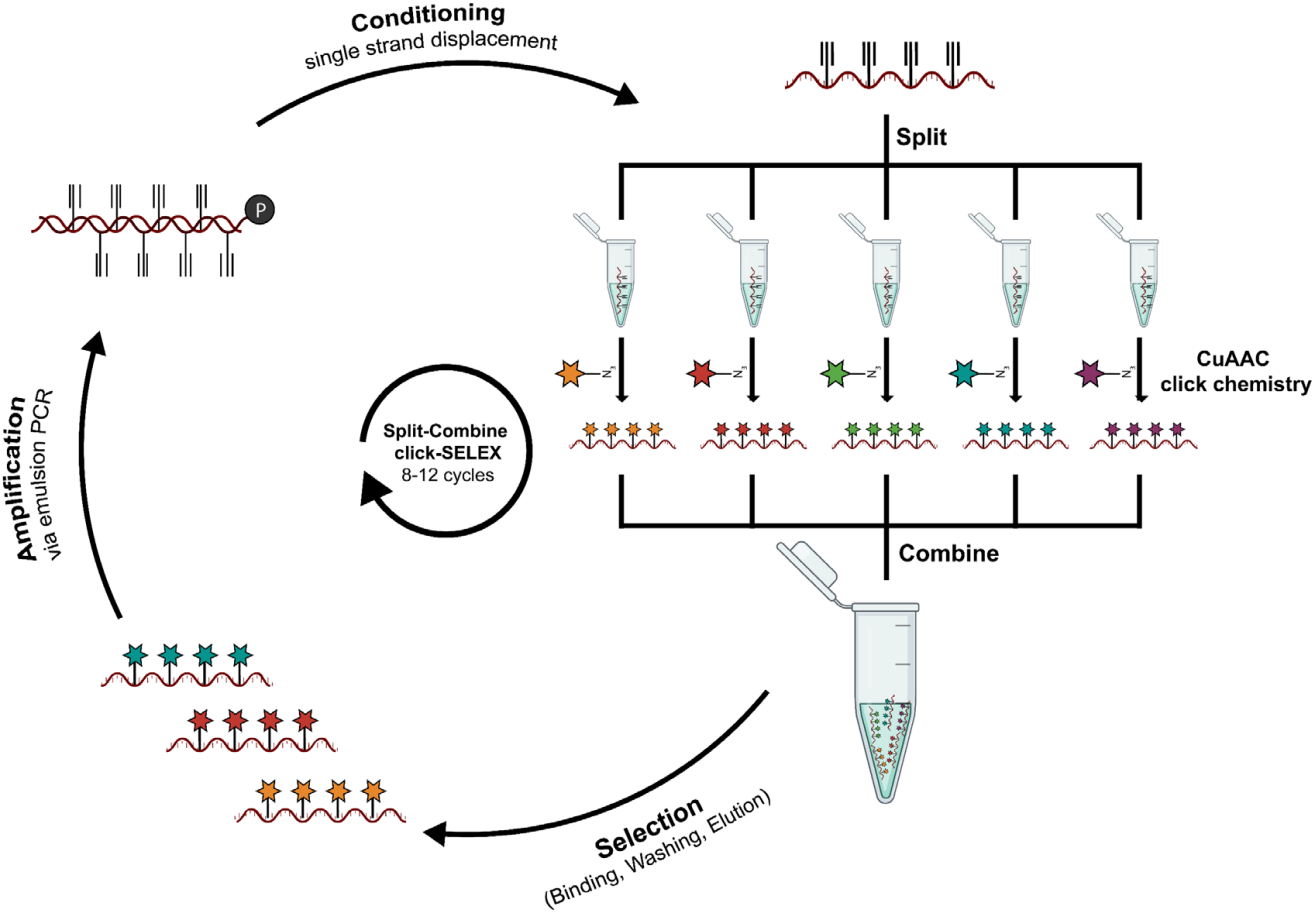
Schematic representation of split‐combine click‐SELEX. The DNA library used in click‐SELEX is synthesized with 5‐ethynyl‐2′‐deoxyuridine (EdU) residues in place of deoxythymidine (dT). The alkyne modifications thereby allow postsynthetic conjugation of azide‐containing functional groups via CuAAC click chemistry. Initially, the synthetic DNA library is divided into several equimolar aliquots (split) and each aliquot is clicked with a different azide modification. After purification, these modified libraries are pooled (combine) and subjected to selection, including target binding, washing of the selection matrix, and elution of bound sequences from the target. Eluted sequences are then amplified by emulsion PCR using an EdU‐containing dNTP mix (excluding dT) and phosphorylated reverse primer. The introduction of the phosphate group during PCR enables digestion of the antisense strand and generation of a single‐stranded library for the next selection cycle. This process is iterated for 8–12 cycles, after which the library is functionalized with each of the modifications separately. This allows probing each modification for binding to the target, leading to the identification of the correct functional group for target binding.^[^ ^4^ ^,^ ^14^ ^,^ ^22^ ^]^

Clickmers are generated through an adapted in vitro selection process termed click‐SELEX (**Figure** **1**), a modified form of the well‐established systematic evolution of ligands by exponential enrichment (SELEX) protocol.^[^ ^4^ ^]^ In click‐SELEX, oligonucleotide libraries incorporating EdU residues^[^ ^5^ ^]^ are chemically diversified through CuAAC click chemistry,^[^ ^6^, ^7^, ^8^ ^]^ enabling the evolution of ligands with enhanced binding affinity, stability, and selectivity toward complex targets such as viral glycoproteins.

We have previously reported the generation of DNA aptamers that bind the CoV2‐S glycoprotein.^[^ ^9^ ^]^ Here, we report the selection and biochemical characterization of clickmers binding to the CoV2‐S glycoprotein, demonstrating the successful enrichment of high‐affinity, chemically diversified clickmers with diagnostic potential. Among the selected candidates, we identified a particularly potent binder, which we further optimized its binding to the Omicron variant through rational truncation. The resulting minimal clickmer, termed T2, retains high‐affinity binding despite reduced sequence length. Importantly and in contrast to the prior developed unmodified DNA aptamers, we show that T2 also binds to the CoV2‐S Omicron variant,^[^ ^10^ ^]^ highlighting the robustness of clickmer‐based recognition even in the context of heavily mutated spike protein variants. These findings underscore the versatility of the click‐SELEX platform and the utility of clickmers as adaptable molecular tools for viral detection and beyond.

## 2 Results

### 2.1 Selection and Characterization of CoV2‐S Binding Clickmers

**Figure 2:**
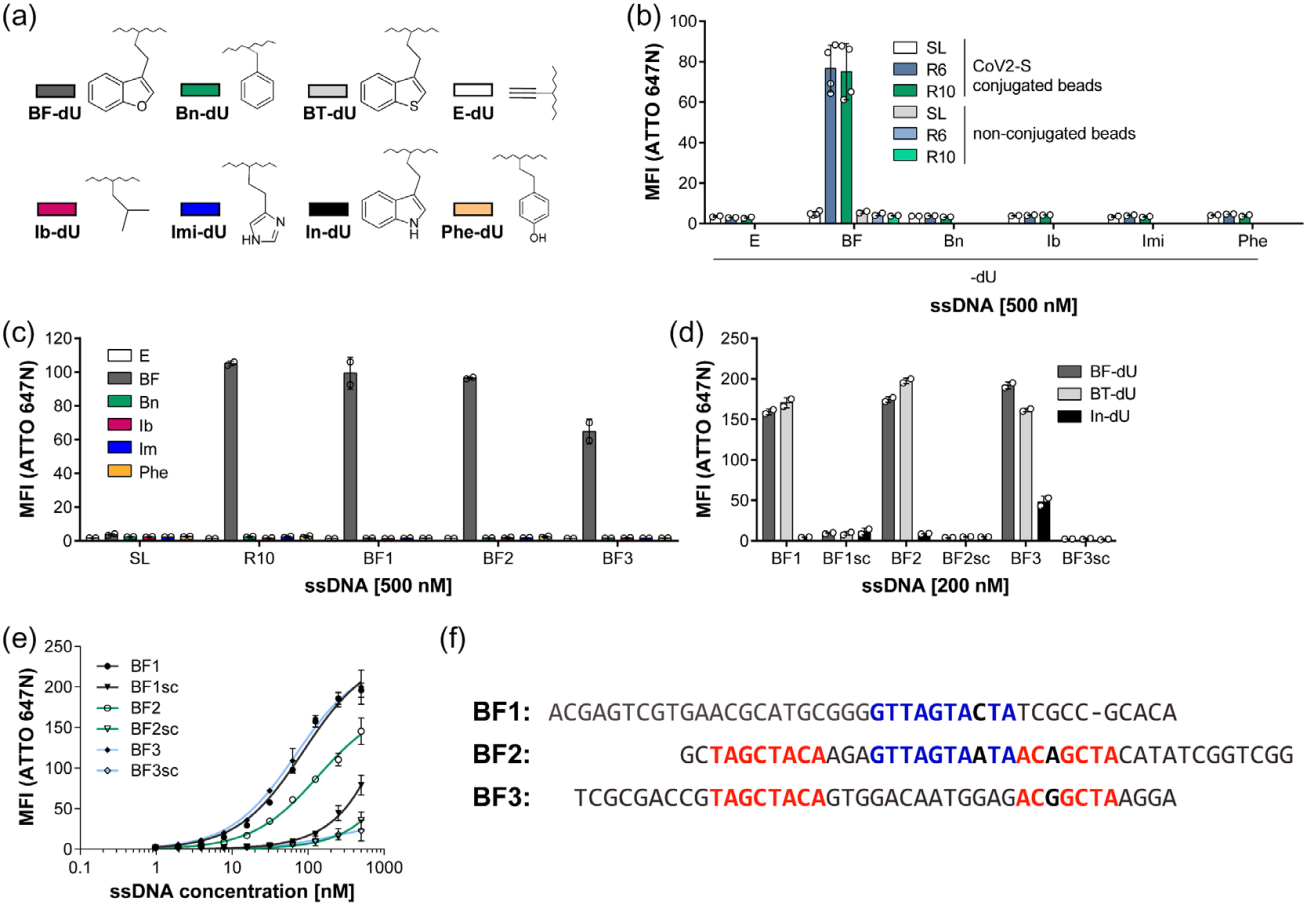
Split‐combine click‐SELEX A targeting SARS‐CoV‐2 spike. a) Chemical structures of the azides used during selection and further characterization. (BF‐dU) 3‐(2‐azidoethyl)‐benzofuran, (Bn‐dU) 1‐(azidomethyl)‐benzene, (BT‐dU) 3‐(2‐azidoethyl) benzo[b]thiophene, (E‐dU) 5‐ethynyl‐dU, (Ib‐dU) 1‐azido‐2‐methylpropane, (Imi‐dU) 4‐(2‐azidoethyl)‐1H‐imidazole, (In‐dU) 3‐(2‐azidoethyl)‐1H‐indole, and (Phe‐dU) 4‐(2‐azidoethyl)‐phenol. b) Flow cytometry‐based interaction assay of enriched DNA libraries from selection cycle 6 (R6) and 10 (R10) in comparison to the SL with unmodified and SARS‐CoV‐2 spike (CoV2‐S) coupled streptavidin beads. Beads were incubated with 500 nM ATTO 647N‐labeled DNA which was unmodified (E‐dU) or click‐modified with benzofuran (BF), benzyl (Bn), isobutyl (Ib), imidazole (Imi), or phenyl (Phe) (*n* = 2, mean ± SD). c) Flow cytometry‐based interaction analysis of selection cycle 10 (R10) in comparison to the SL and sequences BF1, BF2, and BF3 found by NGS. CoV2‐S coupled beads were incubated with 500 nM ATTO 647N‐labeled DNA which was unmodified (E‐dU) or click‐modified with benzofuran (BF), benzyl (Bn), isobutyl (Ib), imidazole (Im), or phenyl (Phe) (*n* = 2, mean ± SD). d) Flow cytometry‐based interaction analysis of sequence 200 nM ATTO 647N‐labeled BF1, BF2, and BF3 in comparison to their scrambled nonbinding controls with CoV2‐S. Sequences were click‐modified either with benzofuran (BF‐dU) or the chemically related modifications benzothiophene (BT‐dU) and indole (In‐dU) (*n* = 2, mean ± SD). e) Binding curves of BF1, BF2, BF3 and scrambled controls obtained by titrating increasing concentrations of oligonucleotide against CoV2‐S protein using flow cytometry (*n* = 2–3, mean ± SD, nonlinear regression using a one‐site specific binding model (GraphPad Prism)). f) Sequence alignment of the enriched clickmer candidates BF1, BF2, and BF3. BF1 and BF2 contain a shared motif (GTTAGTAMTA; motif 1, blue), which is absent in BF3. BF2 and BF3 share a gapped motif (TAGGCTACA…ACRGCTA; motif 2, red) that partially forms a stem. Motifs are highlighted in color with the corresponding nucleotides in bold. Single‐nucleotide mutations within motif regions are shown in black.

To identify clickmers that bind to CoV2‐S (i.e., the trimerized His‐tagged extracellular domain of CoV2‐S, expressed in mammalian cells,^[^ ^9^ ^]^ stabilized in the prefusion conformation),^[^ ^11^ ^]^ we pursued two independent split‐combine click selections (Figure 1),^[^ ^12^ ^]^ dubbed SELEX A and SELEX B, which differ in the set of chemical entities and in the conditions used during the incubation step. SELEX A was pursued to mimic saliva conditions meant to evolve clickmers suitable for diagnostic purposes. Therefore, urea has been added^[^ ^13^ ^]^ to the phosphate buffer and the incubation was done at 25 °C at a pH value of 7.0. In SELEX A Imi‐dU, Ib‐dU, Bn‐dU, Phe‐dU, and BF‐dU were clicked into the library and used in the split‐combine click‐SELEX variant (**Figure** **2**a). In total, 10 selection cycles were performed, applying increasing stringency (Table S1A, Supporting Information) by reducing the amount of target and incubation time as well as by increasing the number of washing cycles. The resulting DNA libraries of selection cycles 6 and 10 were assessed for CoV2‐S binding using flow cytometry. These experiments revealed an increased binding of the DNA population from selection cycles 6 and 10 to CoV2‐S only when modified with BF‐dU. Neither of the other modifications was observed to promote target binding, nor did the starting library (SL) or the enriched libraries in the unclicked state (Figure 2b). Binding to the bead matrix in the absence of CoV2‐S was not detected by the analyzed libraries (Figure 2b). Based on these results, we decided to stop the selection at selection cycle 10 and not to continue with deconvolution cycles, as done previously.^[^ ^14^ ^]^ We performed next‐generation sequencing (NGS) to analyze the DNA population and therefore 10^6^ to 10^7^ sequences were analyzed per selection cycle (Figure S2a, Supporting Information). This analysis shows a strong decrease of the number of unique sequences in the DNA population of selection cycle 5 and leveling at 3.2% throughout the subsequent selection cycles (Figure S2b, Supporting Information). The distribution of nucleotides also changes significantly over the course of the selection cycles. In the 10th selection cycle, a fairly uneven distribution of nucleotides is observed compared to the 1st cycle. (Figure S2c, Supporting Information).

Based on sequence analysis, we chose the representative sequences BF1, BF2, and BF3 for further characterization. BF1 (28.0% in selection cycle 10 population) and BF2 (18.1% in selection cycle 10 population) are among the most enriched sequences, whereas BF3 has a lower overall frequency, peaked in cycle 8 but declined significantly afterward (Figure S2d, Supporting Information). We tested BF1, BF2, and BF3 for interaction with CoV2‐S in dependence of different clicked‐in chemical entities using flow cytometry. All three sequences bind to CoV2‐S when modified with BF‐dU, whereas no interaction could be detected when modified with any of the other modifications used during the enrichment procedure or when unclicked (Figure 2c).

We next used the BF‐dU related clicked‐in moieties In‐dU and BT‐dU, respectively, and tested binding of BF1, BF2, and BF3 when modified accordingly. BF1 and BF2 both showed also binding to CoV2‐S when modified with BT‐dU but not when In‐dU was used (Figure 2d). In contrast, In‐dU modified BF3 still revealed CoV2‐S binding, although to a lesser extent compared to the BF‐dU and BT‐dU modified variants (Figure 2d). We used flow cytometry and BF‐dU modified clickmers BF1, BF2, and BF3 to determine concentration dependent binding of CoV2‐S. These experiments revealed *K*_D_ values of 87 nM (BF1) and 69 nM (BF3), whereas saturation was not achieved for BF2 (*K*_D_ > 135 nM). The corresponding scrambled variants of each clickmer (BF1sc, BF2sc, BF3sc) did not show strong binding to CoV2‐S, but became detectable at higher concentrations, most notably for BF1sc (Figure 2e). A comparative sequence analysis of BF1, BF2, and BF3 revealed two distinct motifs (Figure 2f). BF2 shares motif 1 with BF1 and a gapped motif 2 with BF3, whereas BF1 and BF3 do not share any motif.

**Figure 3:**
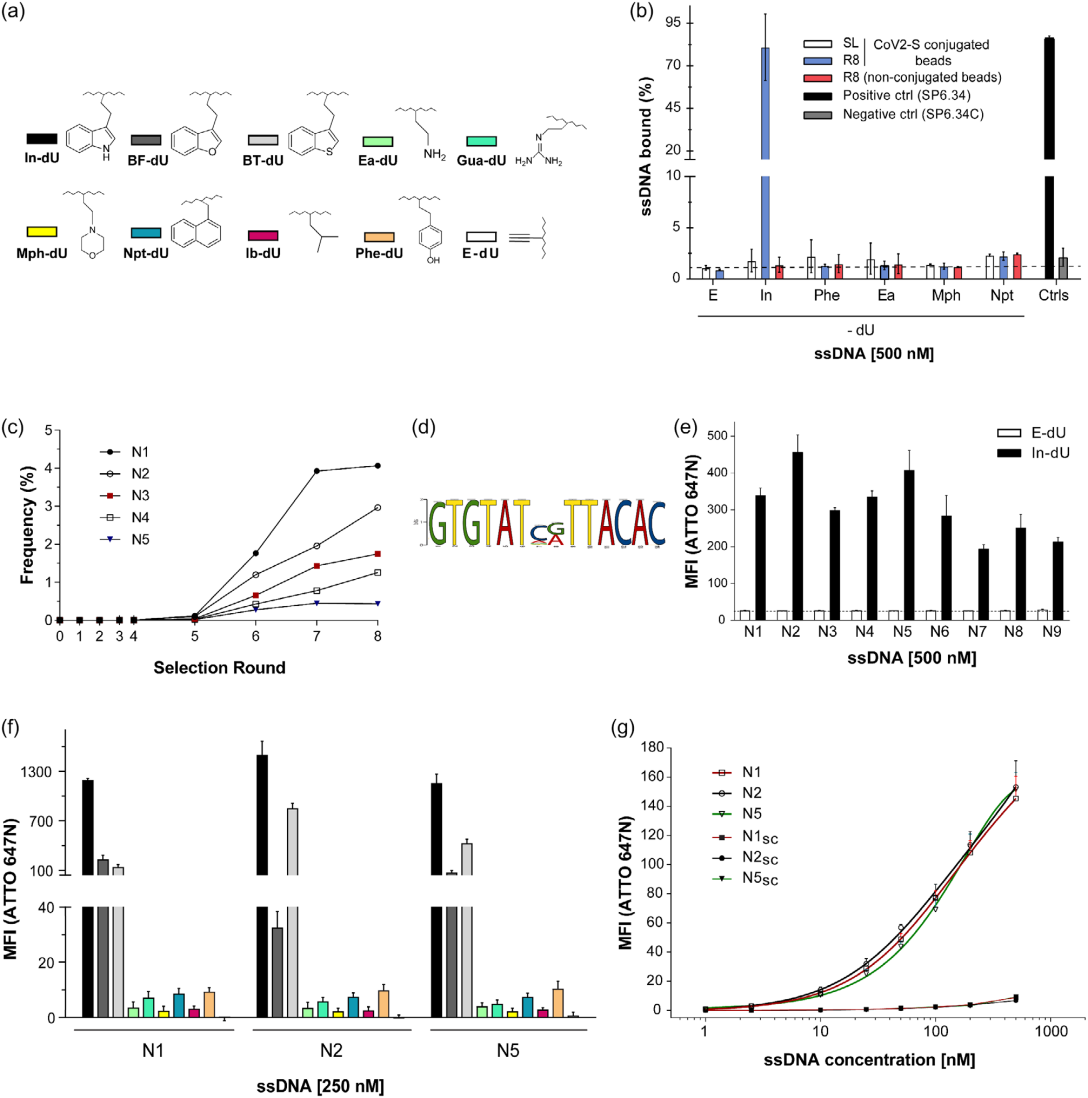
Split‐combine click‐SELEX B targeting SARS‐CoV‐2 spike. a) Chemical structure and annotations of the azides used for selection and characterization. (In‐dU) 3‐(2‐azidoethyl)‐1H‐indole, (BF‐dU) 3‐(2‐azidoethyl) benzofuran, (BT‐dU) 3‐(2‐azidoethyl) benzo[b]thiophene, (Ea‐dU) 2‐azidoethanamine, (Mph‐dU) 4‐(2‐azidoethyl) morpholine, (Npt‐dU) 1 (2 azidomethyl) naphthalene, (Ib‐dU) 1‐azido‐2‐methylpropane, (Phe‐dU) 4‐(2‐azidoethyl) phenol, (Gua‐dU) 2‐(2‐azidoethyl) guanidine hydrochloride, or (E‐dU) unfunctionalized (alkyne). b) Flow cytometer‐based enrichment analysis of round eight in comparison to the SL. 500 nM ATTO 647N‐labeled SL and selection round eight (R8) were incubated with either target‐coupled or nonconjugated magnetic beads. ssDNA was either unmodified (EdU) or click‐modified with indole (In), phenyl (Phe), ethylamine (Ea), morpholine (Mph), naphthalene (Npt) azides. Single bead population was gated and the percentage of ssDNA detected on beads was generated in comparison to beads only. SP6.34 and point mutant SP6.34C were included to validate successful conjugation of target to the beads (*n* = 2 with duplicates, mean ± SD). c) Abundance of several top candidates in each round´s pool obtained via NGS. All the candidates belong to one large family bearing one common motif with five EdU residues. d) Comparative sequence analysis using the MEME Suite^[^ ^28^ ^]^ identified a conserved motif (GTGTATMRTTACAC) shared among the most enriched sequences. e) Interaction study of candidates. 500 nM ATTO 647N‐labeled unmodified (EdU) or indole‐modified (In‐dU) N1–N9 were incubated with COV2‐S HexaPro and the fluorescence of target‐coupled beads was measured on the flow cytometer. The basal mean fluorescence of the target‐coupled beads depicts basal fluorescence detected (*n* = 2 with duplicates for each, mean ± SD). f) Investigation of the click‐dependency of N1, N2, and N5 with various azides. Top three binding clickmers were evaluated for binding properties either functionalized or unmodified with the previously annotated azides (color code as in (a). g) Concentration‐dependent binding curves of top three clickmers: N1, N2, and N5. Series of concentration (1, 2.5, 10, 25, 50, 100, 200, and 500 nM) of each clickmer including their corresponding nonbinding scrambled sequences (N1sc, N2sc, and N5sc) were prepared and incubated in SELEX buffer with SARS‐CoV‐2 spike: (*n* = 3, mean ± SD, nonlinear regression using a one‐site total binding model (GraphPad Prism)).

Phosphate‐buffered saline and similar selection conditions have been successfully used previously to obtain aptamers that performed well in cell culture experiments and in vivo.^[^ ^15^ ^,^ ^16^ ^]^ Thus, SELEX B was conducted in phosphate‐buffered saline as incubation buffer, supplemented with 1 mM Mg^2+^‐ions and at 37 °C. To increase chemical diversity, we selected one modification overlapping (Phe‐dU) with SELEX A and four additional, distinct chemical modifications in SELEX B comprising of In‐dU, Ea‐dU, Mph‐dU, and Npt‐dU (**Figure** **3**a). In total, 8 selection cycles were performed, applying increasing stringency similarly to SELEX A (Table S1B, Supporting Information) by reducing the amount of target and incubation time as well as by increasing the number of washing cycles and the addition of competitors in selection cycles 5–8 (Table S1B, Supporting Information). The DNA library from selection cycle 8 was assessed for CoV2‐S binding by flow cytometry. The results of this experiment showed increasing binding of the DNA library from selection cycle 8 to CoV2‐S but only when modified with In‐dU (Figure 3b). No interaction of the unmodified DNA or the DNA clicked to any of the other residues applied during the selection was detectable (Figure 3b). As for SELEX A, we performed NGS analysis of the DNA populations of all selection cycles. This analysis revealed a strong decrease of the number of unique sequences from selection cycle 5–6 (78.4%–29.2%), which then further declined to 11.7% in selection cycle 8 (Figure S3a, Supporting Information). Plotting the frequency of the top five most enriched sequences (N1–N5) within the cycle 8 pool confirmed the occurrence of an enrichment starting from selection cycle 5 (Figure 3c). Along these lines, the distribution of nucleotides also changed significantly, revealing enrichment of Gs in the first half of the randomized region after 8 selection cycles (Figure S3b, Supporting Information). Based on this sequence analysis, a family sharing a distinct motif having five EdU nucleotides (annotated as Ts) could be identified (Figure 3d). We chose the most enriched sequences of this family (N1–N9) for further characterization. All nine sequences were found to interact with CoV2‐S in a click‐dependent manner (Figure 3e). N1, N2, and N5 were chosen to analyze the click‐dependency using the other chemical entities employed during the selection process, but none of these supported CoV2‐S binding (Figure 3f). The In‐dU analogs used in SELEX A, BF‐dU, and BT‐dU, however did support binding of N1, N2, and N5 to CoV2‐S, but not to the same extent as does In‐dU. By means of flow cytometry we determined concentration dependent binding of In‐dU modified N1, N2, and N5 to CoV2‐S, in which no saturation of signal intensity was observed at concentrations up to 500 nM (Figure 3g). The corresponding scrambled versions of the clickmers (N1sc, N2sc, and N5sc) did not show binding (Figure 3g).

**Figure 4:**
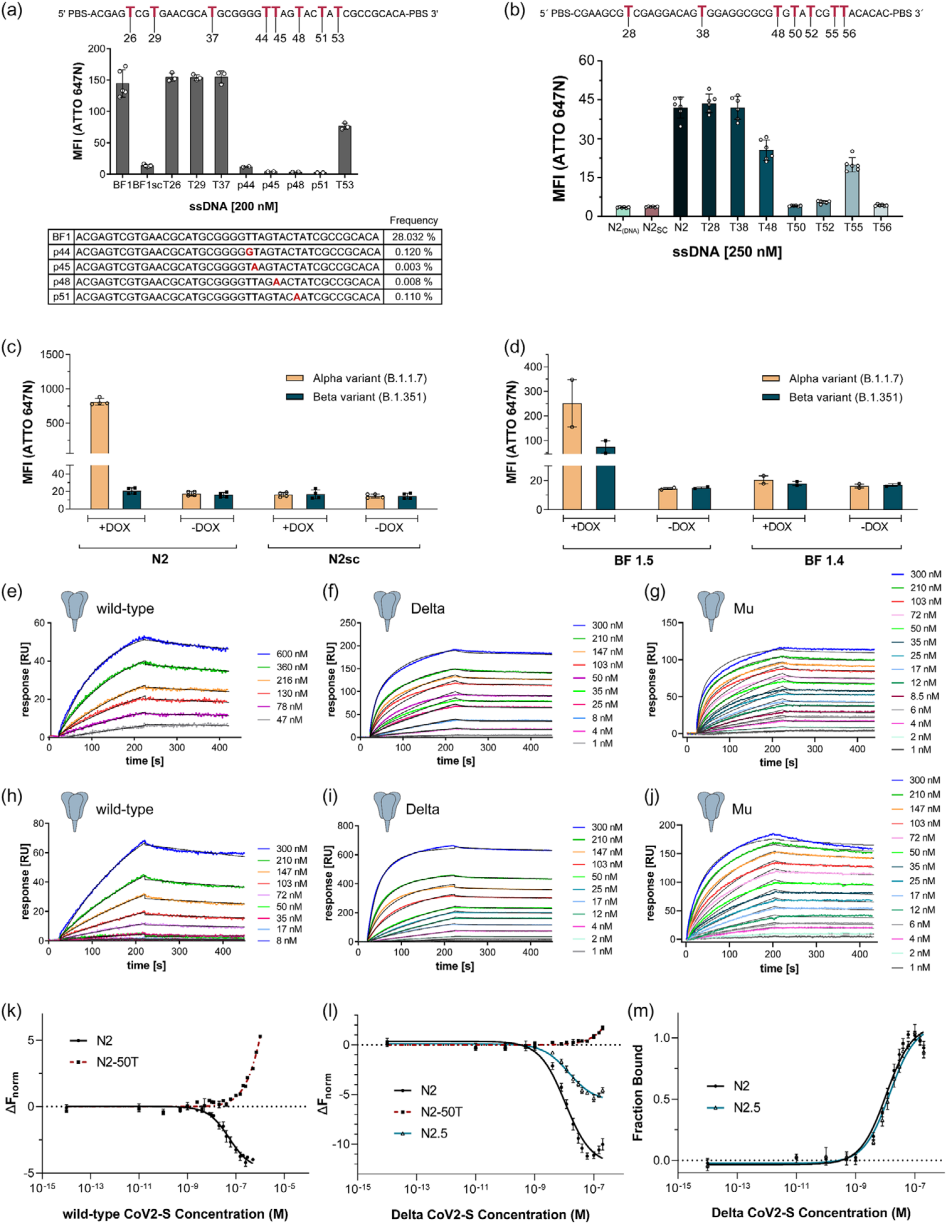
Necessary EdU positions and binding to CoV2‐S variants. Point mutations for all eight EdU positions in a) BF1 and b) N2 were tested to determine the EdU positions crucial for binding. In case of BF1, for positions 44, 45, 48, and 51, point mutants found in the NGS analysis were tested. The table shows the frequency of each point mutant in the BF1‐family. For positions 26, 29, 37, and 53, the EdU was replaced with dT. For N2, seven sequences were synthesized with each corresponding EdU position exchanged to dT one at a time. Binding was tested in a flow‐cytometry‐based interaction assay with CoV2‐S immobilized on magnetic beads using ATTO 647N‐labeled clickmer (*n* = 2–3 duplicates, mean ± SD). PBS, primer binding site. c,d) Cell‐based binding assay of (c) N2 and (d) BF 1.5 via flow cytometry against Alpha and Beta CoV2‐S variants. 50 nM ATTO 647N‐ssDNA was incubated with Flp‐in T‐Rex cells either induced for spike expression (+DOX) or seeded in complete medium lacking doxycycline (‐DOX). After two washes in Dulbecco's phosphate‐buffered saline (DPBS), the cells were analyzed on flow cytometer (*n* = 2 duplicates c) or singlets d), mean ± SD) e–g) surface plasmon resonance (SPR) measurements of clickmer BF1.5 binding to (e) CoV2‐S wild‐type, (f) Delta variant, and (g) Mu variant. h–j) SPR measurements of clickmer N2 binding to (h) CoV2‐S wild‐type, (i) Delta variant, j) Mu variant. Kinetic measurements were done using biotin‐labeled clickmer on streptavidin‐coated sensor chips and CoV2‐S variants at the stated concentrations. The *K*_D_ was determined with TraceDrawer using e,h) a one‐to‐one fit or f,g,i,j) a one‐to‐one two‐state fit (*n* = 2–3). k–m) Microscale thermophoresis (MST) analysis of the N2 and N2.5 equilibrium constant against (k) wild‐type and (l) Delta variant. Thermophoresis was measured with 65% and 40% excitation and MST power, respectively. N2‐50T nonbinding mutant was utilized to contrast out unspecific interactions at high ligand concentration. (m) direct comparison of the affinities between N2 and N2.5 against Delta variant depicted as fraction bound at different concentrations of the ligand (*n* = 4, mean ± SD).

N2 and BF1 clickmers were selected for further investigation to assess the importance of individual EdU positions and to evaluate their binding properties to CoV2‐S glycoprotein from various strains. BF1 contains eight EdU residues and to assess the importance of each residue for CoV2‐S binding, four point‐mutants found in the NGS analysis were tested, each of them having seven BF‐dUs and the 8th EdU replaced by another canonical nucleotide. The point mutants were represented to only 0.003%–0.120% in the BF1 family. For the residual positions we generated four BF1 variants, each of them having seven BF‐dUs and one BF‐dU replaced by thymidine (**Figure** **4**a). Flow cytometry was used to investigate the binding properties of each variant and these data reveal BF‐dU positions 26, 29, and 37 being nonessential. In contrast, replacing BF‐dU at the positions 44, 45, 48, and 51 by thymidine completely abolished CoV2‐S binding and half‐maximal binding compared to BF1 was detected when position 53 was changed (Figure 4a). Based on these results, the variant BF1.5 was opted, which has five BF‐dU residues at positions 44, 45, 48, 51, and 53 but thymidine at positions 26, 29, and 37. Furthermore, to assess the dependence of N2 on individual In‐dU residues, we synthesized seven N2 variants, each carrying a single EdU‐to‐thymidine substitution, thereby rendering the respective site nonreactive to click chemistry. N2 contains seven In‐dU positions, of which two (positions 28 and 38) were found to be dispensable for CoV2‐S binding (Figure 4b). In contrast, substitutions at the remaining positions led to either a strong (positions 50, 52, and 56) or moderate (positions 48 and 55) reduction in binding affinity (Figure 4b). Based on these results, the variant N2.5 was opted.

### 2.2 Characterization of N2 and BF1.5 Binding to CoV2‐S Variants

We next examined the effect of spike protein mutations on the binding capabilities of the two clickmers by testing their interaction with CoV2‐S variants. As recommended in a white paper published by members of the International Society on Aptamers,^[^ ^17^ ^]^ we employed multiple strategies to evaluate clickmer binding to their putative target, thereby increasing the confidence and validity of our measurements. We first employed Flp‐In T‐REx cells expressing Alpha (B1.1.7) or Beta (B1.315) spike variants upon doxycycline induction (+DOX). The N2 clickmer demonstrated specific binding to only Alpha variant when compared to the clicked scrambled control (N2sc), but failed to interact with the Beta variant (Figure 4c). BF1.5 also showed binding to cells expressing the Alpha variant compared to the nonbinding point mutant (BF1.4). In contrast to N2, BF1.5 exhibited moderate binding to the Beta variant (Figure 4d). Only minimal cell‐surface binding was observed in the absence of spike protein (‐DOX), confirming that the identified clickmers display relevant target specificity (Figure 4c,d).

We then aimed to determine the binding affinities of the two clickmers to the CoV2‐S variants. Surface plasmon resonance (SPR) measurements were performed by immobilizing biotinylated clickmer on streptavidin‐coated (SA) sensor chips (Figure 4e–j). Titration with increasing concentrations of wild‐type spike protein yielded equilibrium dissociation constants (*K*_D_) of 27.8 nM for BF1.5 and 40.7 nM for N2, respectively (Figure 4e,h, **Table** **1**). We extended the SPR analysis to assess binding affinities of BF1.5 and N2 against the Delta (B.1.617.2) and Mu (B.1.621) variants. Both clickmers interacted with both of the variants and interestingly exhibited improved affinity toward these two spike variants compared to wild‐type (Figure 4f,g,i,j, Table 1). To validate the affinity constants obtained by SPR, we employed microscale thermophoresis (MST) as another technique to assess the binding of Cy5 labeled N2 to wild‐type and Delta CoV2‐S (Figure 4k,l). Its variant N2.5 was also tested against Delta CoV2‐S (Figure 4l) to confirm that the removal of the two nonessential EdU residues has indeed no significant impact on binding affinity compared to N2 (Figure 4m), as shown previously in Figure 4b. The results from MST measurements were consistent with the SPR data and are summarized in Table 1.

**Table 1:** Summary of equilibrium constants (*K*_D_) obtained for N2, N2.5, and BF1.5 against spike variants using Surface Plasmon Resonance (SPR) and Microscale Thermophoresis (MST).

Clickmer	Spike variant	Temperature [°C]	*K*_D_ [nM]
N2	Wild‐type	37	40.7 ± 0.1 (SPR) 38.8 ± 5.8 (MST)
N2	Delta	37	1.7 ± 0.3 (SPR) 2.3 ± 1.0 (MST)
N2	Mu	37	7.0 ± 4.6 (SPR)
N2.5	Delta	37	3.8 ± 1.7 (MST)
BF1.5	Wild‐type	25	27.8 ± 0.3 (SPR)
BF1.5	Delta	25	1.7 ± 0.2 (SPR)
BF1.5	Mu	25	1.2 ± 0.1 (SPR)

### 2.3 Truncation of N2 Reveals a 31‐Nucleotide Clickmer with Increased Binding to the Omicron CoV2‐S Variant

**Figure 5:**
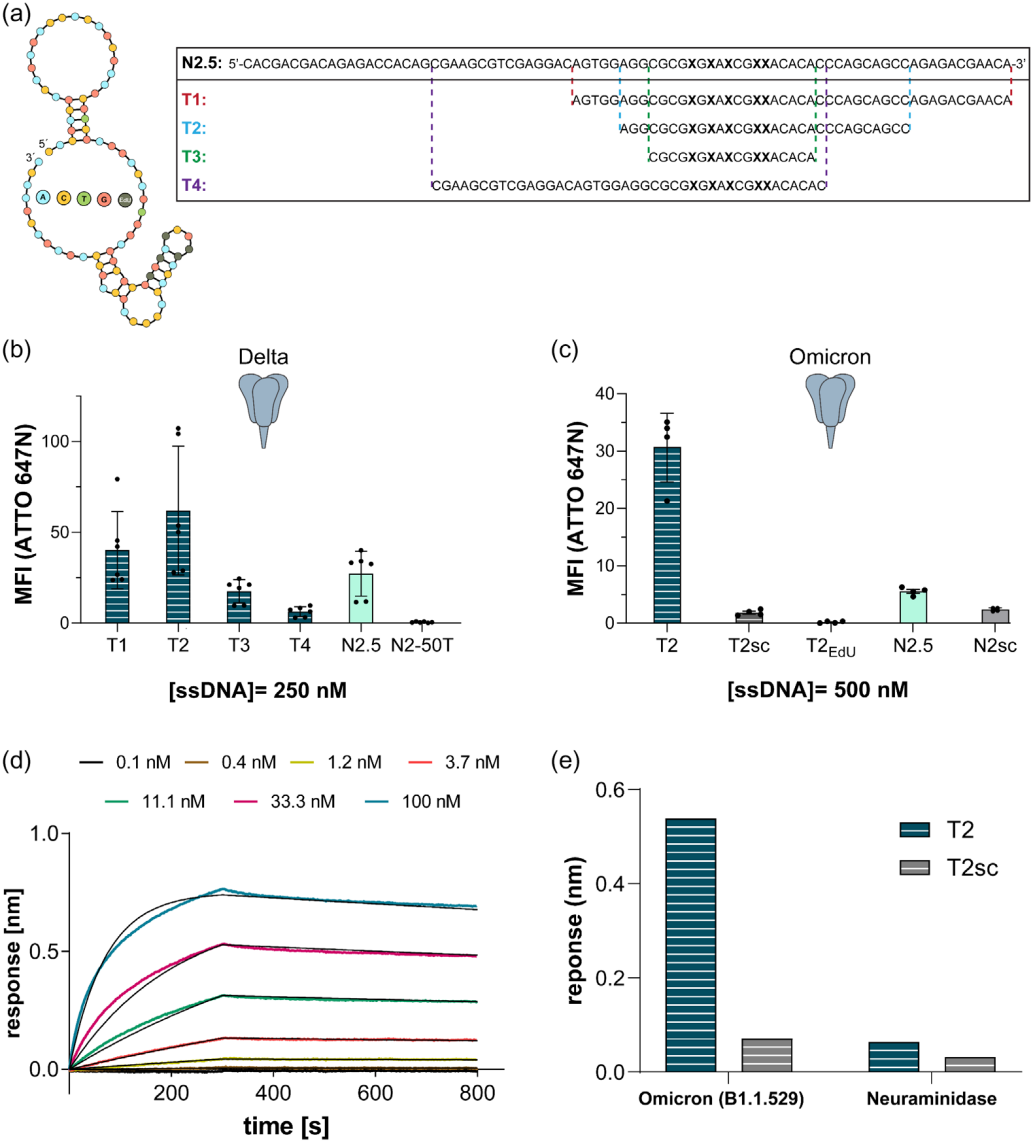
Truncation of N2.5 clickmer and binding characterization of truncate against CoV2‐S variants. a) N2.5 structural prediction by NUPACK as a complete DNA oligonucleotide. The regions of the original clickmer kept for each truncate are highlighted with its corresponding color and dashed lines. T4 is a truncate bearing the 42‐mer random region of N2.5. b) Binding analysis of the truncates. The interaction was analyzed in a flow‐cytometry‐based assay with CoV2‐S Delta immobilized on magnetic beads using 250 nM ATTO 647N‐labeled clickmer and truncates (*n* = 3 duplicates, mean ± SD). N2‐50T (a nonbinding point mutant of N2) was used as negative control. c) Comparison of the binding of N2.5 and T2 against Omicron variant (B.1.1.529). The binding was investigated in a flow‐cytometry based assay with CoV2‐S Omicron immobilized on magnetic beads using 500 nM ATTO 647N‐labeled N2.5 and T2 truncate. As negative controls a scrambled T2 oligo (T2sc), unmodified T2 oligo (T2_EdU_) were used (*n* = 2 duplicates, mean ± SD). d) BLI kinetic analysis. Kinetic measurements were performed using biotin‐labeled clickmer truncate (T2) on streptavidin‐coated sensors and CoV2‐S Omicron variant at the stated concentrations. The *K*_D_ was determined by Prism (GraphPad Prism 5.0) software, using a one‐to‐one binding model. e) BLI based binding analysis of T2 against CoV2‐S Omicron (B.1.1.529) and influenza neuraminidase glycoprotein to test the specificity of the binding by T2 compared to another viral surface protein.

Next, we tested the clickmer ability for binding to the Omicron CoV2‐S variant (B.1.1.529). Initial flow cytometry experiments revealed markedly reduced binding by N2 and a complete loss of affinity for BF1.5 (Figure S4, Supporting Information). In parallel, we observed an increased unspecific interaction of the nonbinding clicked controls against this variant. To improve specificity and potentially enhance accessibility to the Omicron spike protein, several truncated variants of N2.5 were rationally designed based on its predicted secondary structure as a canonical DNA aptamer (**Figure** **5**a) using NUPACK.^[^ ^18^ ^,^ ^19^ ^]^ The predicted structure indicates a structured region comprised of a bulge and hairpin loop that bear the common motif (Figure 3d). Therefore, we generated T1 (47 nt), T2 (31 nt), and T3 (18 nt) truncates of N2.5 that showed complete or partial structural resemblance to N2.5. We also generated another truncate by removing the two 20 nt long primer binding sites (PBS), named T4 that is predicted to partially lose this important structural element (Figure 5a). Using flow cytometry, we initially assessed the binding of each truncated variant to the trimeric Delta spike protein. All three variants (T1, T2, T3) retained binding activity, with T2 exhibiting a notable increase in binding compared to the full‐length N2.5 (Figure 5b). T4 truncate showed a reduction in binding intensity compared to N2.5, consistent with the predicted disruption of the original secondary structure (Figure S5, Supporting Information). Next, we compared the binding of T2 to the Omicron spike protein with that of N2.5. As a nonbinding control, a scrambled version of T2 was generated, referred to as T2sc and we used unmodified T2 to investigate its click‐in dependency. As shown in Figure 5c, the T2 clickmer truncate performs better than N2.5 based on measured mean fluorescence intensity (MFI) only upon indole functionalization. By calculating a fold‐change in fluorescence compared to their scrambled nonbinding controls, T2 shows 16‐fold increase, meanwhile N2.5 shows an increase of 2.3‐fold (Figure 5c).

To further evaluate the binding affinity of the T2 variant to the Omicron spike protein, we performed biolayer interferometry (BLI). Biotinylated T2 was immobilized on SA sensors and exposed to serial dilutions of the trimeric Omicron spike protein. The resulting sensorgrams yielded a dissociation constant (*K*_D_) of 1.3 nM, indicative of a high‐affinity interaction between T2 and Omicron spike (Figure 5d), in agreement with the enhanced binding intensity observed in flow cytometry. To evaluate specificity, T2 was also tested against recombinant neuraminidase (Figure 5e). The response obtained for neuraminidase was markedly lower than that for the Omicron spike, confirming the selective recognition of the spike protein by T2. As expected, the scrambled control T2sc exhibited negligible binding across tested proteins.

## 3 Conclusion

The present study aims to elucidate the identification of disparate clickmers that bind to the CoV2‐S protein under two distinct physiological conditions. A split‐combine click‐SELEX scheme was employed, entailing the concurrent utilization of multiple modifications. In accordance to previous studies,^[^ ^4^ ^,^ ^12^ ^,^ ^14^ ^]^ subsequent analysis revealed that the resultant clickmers exhibited a click‐in specific binding affinity for CoV2‐S among the entities utilized during the click‐SELEX process, namely benzofuran (BF‐dU) or indole (In‐dU). Interaction analysis with closely related functional groups, for example, benzothiophene (BT‐dU), demonstrated the presence of variation potential, offering options for variations of the clickmers’ properties.^[^ ^20^ ^]^ The clickmers revealed discrepancies in specificity with regard to binding to CoV2‐S variants, including Beta, Delta and Omicron. These discrepancies might be attributable to the individual click‐in moieties employed during the enrichment processes or the divergent selection conditions applied. Thymidine nucleotide replacement studies demonstrated that a minimum of five out of eight (clickmer BF1) or seven (clickmer N2) modifications are required to preserve the interaction with the spike protein. We also demonstrated the truncation of the clickmer N2 yielding variant T2 (31 nt). This variant demonstrated a more intense binding affinity to the CoV2‐S variants when compared to the original clickmer (82 nt). This finding suggests that either steric hindrance of the original clickmer sequence, or the adoption of alternative conformations with different interaction properties in the full‐length compared to the truncated version, may underlie the observed binding behavior.^[^ ^16^ ^]^ In line with recent developments such as the MEDUSA platform, which demonstrated that multivalent aptamer assemblies containing chemically modified nucleotides can exploit target geometry and valency to achieve enhanced affinity and selectivity,^[^ ^21^ ^]^ our findings further underscore the potential of chemical modifications to fine‐tune binding specificity and performance. The described clickmers represent promising candidates for both diagnostic development and potential therapeutic exploration. This work highlights the adaptability of the click‐SELEX approach, particularly in urgent contexts where rapid response is critical, and illustrates its potential to deliver sensitive detection probes for immediate pandemic or viral outbreak control.

## 4 Experimental Section

4.1

4.1.1

##### Click Chemistry

The click reaction was carried out as previously described in Ref. [4]. Briefly, a Cu(I)‐catalyst solution was freshly prepared by mixing 100 mM sodium ascorbate (Roth, Karlsruhe, Germany), 1 mM CuSO_4_ (baseclick, Neuried, Germany), and 4 mM THPTA (baseclick, Neuried, Germany) in deionized water to a final volume of 100 µL. For the CuAAC reaction, 10 µL of 10 mM azide in DMSO (quality control data published in previous studies), 10 µL of 100 mM phosphate buffer (6.15% (*v*/*v*) 1 M K2 HPO4, 3.85% (*v*/*v*) 1 M KH2 PO4, pH 7.0), 10 µL of the catalyst solution, and 70 µL of the DNA solution in Milli‐Q water were combined and incubated at 37 °C for 45 min with shaking at 650 rpm. Postreaction, samples were purified using the NucleoSpin Gel and PCR Clean‐up Kit (Macherey–Nagel, Düren, Germany) according to the manufacturer's instructions, employing a 4:1 NTC buffer‐to‐sample ratio. DNA was eluted in 20 µL Milli‐Q water with extended incubation (2 min) and multiple elutions to improve recovery.

All azide compounds used in this study are identical to those employed in our previous studies,^[^ ^14^ ^,^ ^20^ ^,^ ^22^ ^]^ where detailed quality control data are reported. The indole azide was resynthesized during the course of this study according to previously published work;^[^ ^23^ ^]^ its NMR spectrum is provided in (Figure S6, Supporting Information).

##### Emulsion PCR Amplification of the Library

To amplify eluted aptamer pools, ePCR^[^ ^24^, ^25^, ^26^ ^]^ was performed in a biphasic system comprising a 150 µL aqueous phase and a 600 µL oil phase (4:1, *v*/*v*; 73% TEGOSOFT, 20% mineral oil, 7% ABIL WE). A 450 µL master mix was prepared, supplemented with freshly prepared bovine serum albumin (BSA) (100 mg mL^−1^), and aliquoted. Tungsten carbide beads (Qiagen, Hilden, Germany) were added to each reaction tube, and emulsification was achieved by shaking for 40 s at 15 Hz, followed by a 10 s rest and another 10 s agitation using a tissue lyzer. Up to 100 µL of each emulsion was transferred to a 96‐well PCR plate and amplified using Veriti thermal cycler (Applied Biosystems, Thermo Fisher Scientific, Waltham, MA, USA) using the following thermal protocol: initial denaturation 2 min at 90 °C; 30 s at 90 °C, 30 s at 58 °C, and 30 s at 72 °C; final extension 2 min at 72 °C; hold at 10 °C. Postamplification, emulsions were pooled and centrifuged (12,000 × g, 10 min), and the lower colloidal phase was recovered. Emulsions were disrupted by adding 300–500 µL phenol/chloroform/isoamyl alcohol (PCI, 25:24:1), vortexing (≈1 min), and centrifugation (16,000 × g, 3 min). The aqueous phase was collected and purified using the NucleoSpin Clean‐Up kit (Macherey–Nagel), employing a 4:1 NTC buffer‐to‐sample ratio. DNA was eluted in 20 µL Milli‐Q water with extended incubation (2 min) and multiple elutions to improve recovery.

##### Agarose Gel Electrophoresis

Agarose LE (Genaxxon, Ulm, Germany) was used to prepare 4% agarose gels prestained with ethidium bromide (Roth). 1 µL 6x DNA Loading Dye (Thermo Scientific, Waltham, MA, USA) was mixed with 5 µL of dsDNA products and loaded on the gel. As reference, 4 µL of GeneRuler Ultra Low Range DNA Ladder (Thermo Scientific) were loaded. A Genoplex system (VWR, Darmstadt, Germany) was used for visualization of the stained dsDNA.

##### Split‐Combine Click‐SELEX Targeting SARS‐CoV‐2 Spike (CoV2‐S, WT HexaPro)

Two split‐combine selections were performed targeting CoV2‐S HexaPro differing in the selection conditions and the chemical entities used for the click modification of the library. The design of **SELEX A** was based on the selection of aptamers for in vitro diagnostics and was therefore performed in saliva‐like buffer at 25 °C. **SELEX B** was designed to select aptamers for therapeutic purposes and was therefore performed in phosphate‐buffered saline at 37 °C.

For the first selection cycle 100 pmol of M2 library and click‐competitor were click modified independently with Imi‐dU, Ib‐dU, Bn‐dU, Phe‐dU, BF‐dU (**SELEX A**) or In‐dU, Ea‐dU, Phe‐dU, Mph‐dU, Npt‐dU (**SELEX B**). Modified DNA sequences were pooled together and incubated with CoV2‐S on magnetic beads in 1x SELEX buffer (**SELEX A**: Saliva‐like buffer^[^ ^12^ ^]^ (34.7 mM Na_2_HPO_4_*2 H_2_O; 11.3 mM NaH_2_PO_4_*2 H_2_O; 4 mM KCl, 3 mM urea; 3 mM MgCl_2_; 1 mg ml^−1^ BSA; 0.05% Tween‐20; adjusted to pH 7.0), **SELEX B**: 3.93 mM Na_2_HPO_4_*2 H_2_O; 1.47 mM KH_2_PO_4_* H_2_O; 137 mM NaCl; 2.68 mM KCl; 1 mM MgCl_2_; 1 mg ml^−1^ BSA; 0.1% Tween‐20, 0.1 mg ml^−1^ Salmon sperm DNA, adjusted to pH 7.4) according to the temperature and incubation conditions in Table S1A,B, Supporting Information (**SELEX A**: 25 °C, **SELEX B**: 37 °C). Depending on the selection scheme, click‐competitors containing similar chemical entities as the library used for SELEX were added to the mixture to suppress nonspecific interactions mediated by the chemical moieties more effectively than salmon sperm DNA and BSA only. Beads were washed with 1x SELEX buffer (without BSA and salmon sperm DNA for **SELEX B**) according to Table S1A,B, Supporting Information and the DNA was eluted by incubation for 5 min at 95°C in 80 µL. The supernatant was used as template for emulsion PCR in a total volume of 750 µL aqueous phase. Purified dsDNA with a 5′‐phosphate at the reverse strand was incubated with lambda‐exonuclease (Thermo Fisher) according to the manufacturer's protocol for 20 min at 37 °C and 650 rpm to selectively digest the 5′‐phosphorylated strand. Samples were purified using NucleoSpin Gel and PCR clean‐up kit (Macherey–Nagel) according to the manufacturer's instructions. Purified ssDNA was aliquoted to five samples, which were click‐modified along with 100 pmol click‐competitor independently with the respective azides. For the next selection cycles several steps were modified to increase the selection pressure: 1) decrease in the amount of target protein immobilized per bead (only for **SELEX A**); 2) decrease in the amount of target protein; 3) decrease in incubation time with the target protein; 4) increase in washing time and steps; and 5) addition of competitors (**SELEX A**: Salmon sperm DNA from cycle 7, **SELEX B**: click‐competitor from cycle 5). A list of the SELEX details can be found in Table S1A,B, Supporting Information.

##### Next Generation Sequencing

The SL and stored pools from each SELEX round were analyzed via NGS on the Illumina NextSeq 500 platform. Sample preparation was performed using the TruSeq DNA PCR‐Free LT kit (Illumina, San Diego, CA, USA) following a published protocol.^[^ ^27^ ^]^ To incorporate unique index nucleotides, ePCR was employed with the same conditions described previously, using multiple distinct indexed primers to barcode individual SELEX pools for multiplexed sequencing. PCR products were purified using the NucleoSpin Clean‐Up kit (Macherey–Nagel), pooled in equal amounts to yield a total of 2 µg ssDNA, and subjected to adapter ligation as per the manufacturer's instructions. Final libraries were purified by agarose gel extraction. Sequence data were analyzed using the in‐house AptaNext pipeline and the MEME Suite.^[^ ^28^ ^]^ Dominant sequence superfamilies emerged in final rounds, from which the most abundant variants were selected for downstream binding analysis via flow cytometry.

##### Constructs, Protein Expression and Purification

All spike constructs are based on SARS‐CoV‐2‐S‐HexaPro (plasmid kindly provided by Jason McLellan, The University of Texas at Austin, USA), the prefusion‐stabilized ectodomain of the S protein carrying on the C‐terminus a trimerization motif, a HRV 3C cleavage site, 8xHis and TwinStrep tags.^[^ ^11^ ^]^ Proteins denoted by ΔHis or ΔST do not contain the respective tag but are otherwise identical to their tag‐containing counterparts. Cloning was done as described in Ref. [5]. Coding sequences of all constructs were verified by Sanger sequencing (Eurofins, Ebersberg, Germany). The CoV‐2 S proteins were expressed in FreeStyle 293F cells and purified exactly as described in Ref. [5].

##### Flow Cytometry‐Based Interaction Analysis

To facilitate flow cytometry‐based interaction analysis of oligonucleotides to the target protein using FACSCanto II cytometer (BD Biosciences, Heidelberg, Germany), the SELEX library was fluorescently labeled by using a 5′‐modified ATTO 647N forward primer (Ella Biotech, Fürstenfeldbruck, Germany) during ePCR amplification. Monoclonal sequences (Table S2, Supporting Information) were synthesized by Ella Biotech with an ATTO 647N fluorophore conjugated at the 5′ end.

For bead‐based assays, 1 µL of protein‐conjugated Dynabeads (M‐280 streptavidin or His‐Tag isolation and pulldown; Invitrogen) was incubated with ATTO 647N‐labeled ssDNA (100–500 nM) in 10 µL of SELEX buffer (including appropriate competitors) for 20 min at 25 or 37 °C under gentle agitation (800 rpm). The beads were washed three times, resuspended in 200 µL of SELEX buffer, and analyzed by flow cytometry (50,000 events). The mean ATTO 647N fluorescence intensity in the APC‐A channel was recorded, and the percentage of beads bound by labeled ssDNA was determined by gating on target beads only.

For cell‐based binding assays, Flp‐In T‐REx‐293 stable cell lines with doxycycline‐inducible surface expression of CoV2‐S protein variants were kindly provided by Prof. Dr. Florian I. Schmidt (University of Bonn, Germany). Cells were cultured in DMEM supplemented with 10% fetal calf serum (FCS), 4 µg mL^−1^ blasticidin (Gibco, Thermo Fisher Scientific, Waltham, MA, USA), and 50 µg mL^−1^ hygromycin B (Invitrogen). For binding experiments, cells induced with 1 µg mL^−1^ doxycycline (+DOX) or maintained in medium without doxycycline (‐DOX) were incubated with 50 nM ATTO 647N‐labeled ssDNA in DPBS supplemented with 1 mM MgCl_2_, 1 mg mL^−1^ BSA, and 0.2 mg mL^−1^ salmon sperm DNA (Invitrogen) for 20 min at 37 °C. After incubation, cells were detached using 0.25% Trypsin‐EDTA (Gibco), pelleted by centrifugation at 200 g, and washed twice with DPBS to remove unbound oligonucleotides. The resulting cell pellets were resuspended in DPBS containing 0.5% BSA and analyzed by flow cytometry (50,000 events recorded per sample). ATTO 647N fluorescence was detected in the APC‐A channel and quantified as MFI.

##### Surface Plasmon Resonance

SPR experiments were conducted using a Reichert SR7000DC dual‐channel instrument (Buffalo, NY, USA). Prior to use, all buffers were filtered through 0.22 µm membranes. Two different running buffers were employed depending on the SELEX variant (**SELEX A** buffer (saliva‐like composition): 34.7 mM Na_2_HPO_4_ · 2H_2_O, 11.3 mM NaH_2_PO_4_ · 2H_2_O, 4 mM KCl, 3 mM urea, 3 mM MgCl_2_, 1 mg mL^−1^ BSA, 0.05% Tween‐20, pH adjusted to 7.0; **SELEX B** buffer: 3.93 mM Na_2_HPO_4_ · 2H_2_O, 1.47 mM KH_2_PO_4 _· H_2_O, 137 mM NaCl, 2.68 mM KCl, 1 mM MgCl_2_, 1 mg mL^−1^ BSA, 0.1% Tween‐20, 0.1 mg mL^−1^ salmon sperm DNA, pH adjusted to 7.4). A regeneration buffer consisting of 100 mM EDTA, 50 mM NaCl, and 0.01% SDS was used between binding cycles. Biotinylated aptamers (50 nM in 0.5 M NaCl) were immobilized on SA sensor chips (SCR SAD500L, XanTec bioanalytics GmbH, Germany) at 25 °C and a flow rate of 15 µL min^−1^ for 1.5 min, following the manufacturer's instructions. A nonbinding control aptamer was immobilized on the reference channel, matched to the same response level as the analyte‐binding aptamer. Protein analytes were injected at 25 °C (for SELEX A‐derived sequences) or 37 °C (for SELEX B‐derived sequences) at a flow rate of 35 µL min^−1^ for 200 s, followed by a dissociation phase of 240 s. Binding affinities against wild‐type CoV2‐S were calculated using TraceDrawer 1.9.2 (Ridgeview Instruments AB) employing a one‐to‐one binding model fit. For CoV2‐S variants (Mu, Delta), a one‐to‐one two‐state binding model provided a better fit.

##### Microscale Thermophoresis

MST experiments were performed using a Monolith NT.115 instrument (NanoTemper Technologies, Munich, Germany). A series of 10 µL binding solutions was prepared by mixing 20 nM of ATTO 647N‐labeled indole‐functionalized clickmer or nonbinding control with serial dilutions of either wild‐type (1 µM to 10 nM) or Delta (300–10 nM) CoV2‐S spike protein in MST buffer consisting of 20 mM HEPES/NaOH (pH 7.8), 150 mM NaCl, 3 mM KCl, 3 mM MgCl_2_, and 10 µM BSA. After mixing, samples were loaded into standard‐treated capillaries (Monolith NT.115 Series, NanoTemper) and equilibrated in the instrument chamber at 37 °C for 10 min. Measurements were carried out at 37 °C using 65% LED power (red excitation), with IR‐laser set to 40% power. A 1 s cold phase was followed by a 20 s hot phase. Each MST run included four replicates for the clickmer and two for the nonbinding control. Data were analyzed using NanoTemper's MO. Affinity Analysis software and equilibrium constants (*K*_D_) were determined by fitting the thermophoretic dose response curves using a one‐to‐one binding *K*_D_ model (the law of mass action).

##### Bio‐Layer Interferometry

BLI were conducted using the Octet RED96 system (ForteBio, Sartorius, Göttingen, Germany). All measurements were carried out in black, flat‐bottom 96 well plates (Grenier, Frickenhausen, Germany) with orbital shaking at 1000 rpm. Biotinylated T2 aptamer or the scrambled control sequence T2sc were diluted to 80 nM in binding buffer (Phosphate‐buffered saline supplemented with 0.02% of Tween‐20 and 0.1 mg mL^−1^ of BSA) and immobilized onto SA sensors (Octet SA Biosensors, Sartorius) until a response of 0.5 nm was reached. A baseline signal was recorded in buffer prior to the association phase. For kinetic measurements, eight aptamer‐loaded sensors were exposed to 1:3 serial dilutions of trimeric Omicron spike protein (ranging from 100–0 nM), preincubated with 50 nM of click‐competitor, for 300 s. Dissociation was monitored by transferring the sensors to buffer‐only wells for 500 s. Sensor regeneration was performed by three cycles of sequential dipping into 500 mM phosphoric acid followed by buffer, each for 5 s.

To assess specificity, aptamer‐loaded sensors were exposed to a single concentration (50 nM) of Omicron spike protein (B1.1.529) and recombinant neuraminidase protein, each preincubated with 50 nM of click‐competitor. Sensorgrams were reference‐subtracted, aligned to the dissociation phase, and globally fitted to a 1:1 binding model using the Octet and GraphPad Prism 5.0 software.

## 5 Conflict of Interest

The authors declare no conflict of interest.

## Supporting information

Supplementary Material

## Data Availability

The data that support the findings of this study are available from the corresponding author upon reasonable request.
