# Supplementary material for: Selection and Characterization of SARS‐CoV‐2 Spike Binding Clickmers
Source: Chembiochem. 2026 Jan 9;27(1):e202500733. doi: 10.1002/cbic.202500733 (PMC12789893; doi:10.1002/cbic.202500733)
Supplement: Supplementary file 1 — Supplementary Material [file CBIC-27-e202500733-s001.pdf]

Supporting information:

Table S1: Overview of of the split-combine click-SELEX A and B conditions using M2 library targeting CoV2-S.

A)

SELEX cycle	Non-conjugated beads [µL]	CoV2-S beads [µL]	Estimated CoV2-S HexaPro [pmol]	Incubation at 25°C [min]	Wash volume [µL]	Washing steps at 25°C, 800 rpm	Competitor [pmol/azide]	Salmon sperm DNA [mg/mL]	ePCR cycles
**1**	100	143	100	60	200	1x 30 s, 2x 3 min	100	------	26
**2**	100	143	67	45	200	1x 30 s, 2x 5 min	100	------	26
**3**	100	143	67	30	200	1x 30 s, 2x 5 min	100	------	28
**4**	100	143	60	30	200	1x 30 s, 2x 5 min	100	------	28
**5**	100	143	60	20	200	1x 30 s, 3x 5 min	100	------	26
**6**	100	143	60	20	200	1x 30 s, 4x 5 min	100	------	22
**7**	100	86	30	20	200	1x 30 s, 4x 5 min	100	0.1	18
**8**	100	86	30	15	200	1x 30 s, 2x 5 min, 1x 10 min	100	0.5	15
**9**	100	35	10	15	200	1x 30 s, 2x 5 min, 1x 10 min	100	0.5	13
**10**	100	35	10	15	200	1x 30 s, 2x 5 min, 1x 10 min	100	0.5	11

B)

SELEX cycle	Negative selection step(s)	Non-conjugated beads [µL]	CoV2-S Beads [µL]	Estimated CoV2-S HexaPro [pmol]	Incubation at 37°C [min]	Washing steps at 37°C, 800 rpm	Wash volume [µL]	Pooled Competitor to library amount ratio	ePCR cycles (Cycles of additional ePCR)
**1**	1	100	285	100	60	3× 30 s	200	------	26
**2**	1	100	285	100	60	3× 30 s	200	------	26 (10)
**3**	1	100	285	100	40	3× 2 min	200	------	32 (8)
**4**	2	100	143	50	20	4× 2 min	200	------	32 (12)
**5**	2	100	143	50	20	4× 5 min	200	1:1	38 (15)
**6**	2	100	72	25	20	4× 5 min	400	2:1	38
**7**	2	100	36	12.5	20	4× 5 min	400	3:1	38
**8**	2	100	36	12.5	20	4× 5 min	400	3:1	38

Table S2: Oligonucleotide sequences. All oligonucleotides were synthesized using TIPS-protected EdU, represented as "X" in the sequences.

Name	Sequence (5' → 3')
**M2 library**	CACGACGACAGAGACCACAG-N_{42}-CCAGCAGCCAGAGACGAACA<br>(N = dA:dC:dG:EdU = 1:1:1:0.7)
**M2- forward primer**	CACGACGACAGAGACCACAG
**M2- reverse primer**	Phosphate-TGTTCGTCTCTGGCTGCTGG
**BF1**	CACGACGACAGAGACCACAGACGAGXCGXGAACGCAXGCGGGGXXAGXACXAX<br>CGCCGCACACCAGCAGCCAGAGACGAACA
**BF1sc**	CACGACGACAGAGACCACAGACGAGXCGXGAACGCAXGCGGGGXXAGXACAAX<br>CGCCGCACACCAGCAGCCAGAGACGAACA
**BF2**	CACGACGACAGAGACCACAGGCXAGCXACAAGAGXXAGXAAXAACAGCXACAXAX<br>CGGXCGGCCAGCAGCCAGAGACGAACA
**BF2sc**	CACGACGACAGAGACCACAGCGAACAAXXGXGXACGGAGXXAAXACXAAAXACXC<br>GCGGCAGCCAGCAGCCAGAGACGAACA
**BF3**	CACGACGACAGAGACCACAGAXCGCGACCGXAGCXACAGXGGACAAXGGAGACG<br>GCXAAGGACCAGCAGCCAGAGACGAACA
**BF3sc**	CACGACGACAGAGACCACAGGCACAAACCCGGXGXCAGGXAGGAAAAGXAGCGAG<br>XGGCXACCCAGCAGCCAGAGACGAACA
**BF1.5**	CACGACGACAGAGACCACAGACGAGTCGTGAACGCATGCGGGGXXAGXACXAXCG<br>CCGCACACCAGCAGCCAGAGACGAACA
**BF1.4**	CACGACGACAGAGACCACAGACGAGTCGTGAACGCATGCGGGGXXAGXACAAXCG<br>CCGCACACCAGCAGCCAGAGACGAACA
**N1**	CACGACGACAGAGACCACAGGCGCGCGGAGXGGGXGXAXCAXXACACAAGG<br>CGGAGXGAGGACCAGCAGCCAGAGACGAACA
**N2**	CACGACGACAGAGACCACAGCGAAGCGXCGAGGACAGXGGAGGCGCGXGXA<br>XCGXXACACAC CCAGCAGCCAGAGACGAACA
**N2.5**	CACGACGACAGAGACCACAGCGAAGCGTCGAGGACAGTGGAGGCGCGXGXA<br>XCGXXACACAC CCAGCAGCCAGAGACGAACA
**N3**	CACGACGACAGAGACCACAGCAAGGGGGCAGGGAGACGCCGXGXAXAGXXA<br>CACCACAAGAACCAGCAGCCAGAGACGAACA
**N4**	CACGACGACAGAGACCACAGGACCACCGAAGGCAGXCGXGGAGXGXAXCGX<br>XACACGCAGGACCAGCAGCCAGAGACGAACA
**N5**	CACGACGACAGAGACCACAGCAACGGCGGCCCACAGGAGGXAGXGAGGXGX<br>AXGGXXACACGCCAGCAGCCAGAGACGAACA
**N6**	CACGACGACAGAGACCACAGAAACGAGCGGCGGGGXGXAXCAXXACACAAC<br>XGAGCAAGGGACCAGCAGCCAGAGACGAACA
**N7**	CACGACGACAGAGACCACAGACCACAGCAACGGCGGCGAGGXAGXGAGGXG<br>XAXGGXXACACGCCAGCAGCCAGAGACGAACA
**N8**	CACGACGACAGAGACCACAGCACGGACGAGXGXCGACGACGXGXAXCGXXA<br>CACGCACCGCCCCAGCAGCCAGAGACGAACA
**N9**	CACGACGACAGAGACCACAGGACACGGGCAAAGAXAGXGXAXCAXXACACC<br>ACGGACGCGGACCAGCAGCCAGAGACGAACA
**N1sc**	CACGACGACAGAGACCACAGGGAGXGGAXXCXAXGXGCAGCGAGAGCGGAC<br>GCAGAGCGXGACCAGCAGCCAGAGACGAACA
**N2sc**	CACGACGACAGAGACCACAGAGXGGCXGXGCGGXGACGXGXGAAGGCAACG<br>CAXGCACAACCCCAGCAGCCAGAGACGAACA
**N5sc**	CACGACGACAGAGACCACAGAXAGCACACGXGCGCAXGAGGXCGAXCGGCG<br>GAXCGAGAXGGCCAGCAGCCAGAGACGAACA
**T1**	AGTGGAGGCGCGXGXAXCGXXACACACCCAGCAGCCAGAGACGAACA
**T2**	AGGCGCGXGXAXCGXXACACACCCAGCAGCC
**T3**	CGCGXGXAXCGXXACACA
**T4**	CGAAGCGTCGAGGACAGTGGAGGCGCGXGXAXCGXXACACAC
**T2sc**	GCCGAACCGCAAGXCACGXXCAGXCCGGCAX

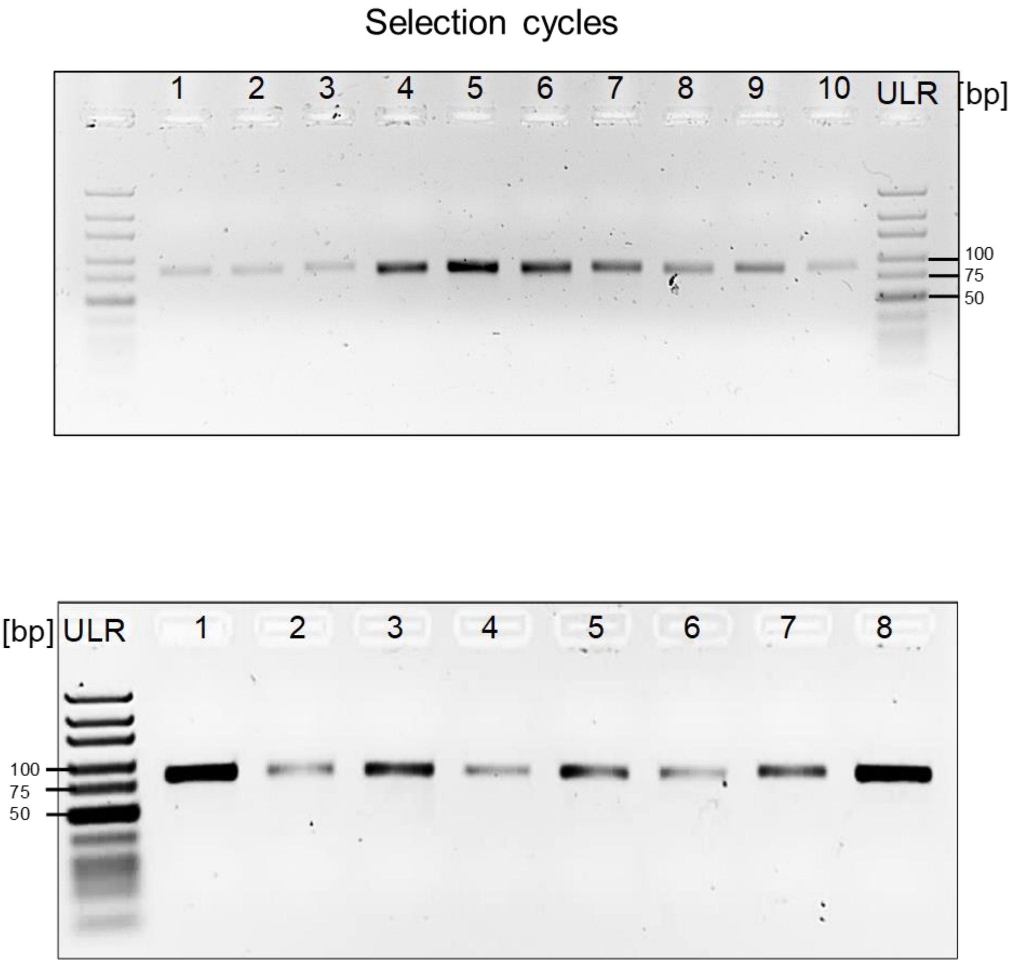

Figure S1: Agarose gel electrophoresis analysis of the ePCR products obtained from SELEX A (top, cycles 1-10) and SELEX B (bottom, cycles 1-8). ULR: GeneRuler Ultra Low Range DNA ladder. For this selection the M2 library was used (dsDNA: 82 bp with seven EdU modifications in the random region).

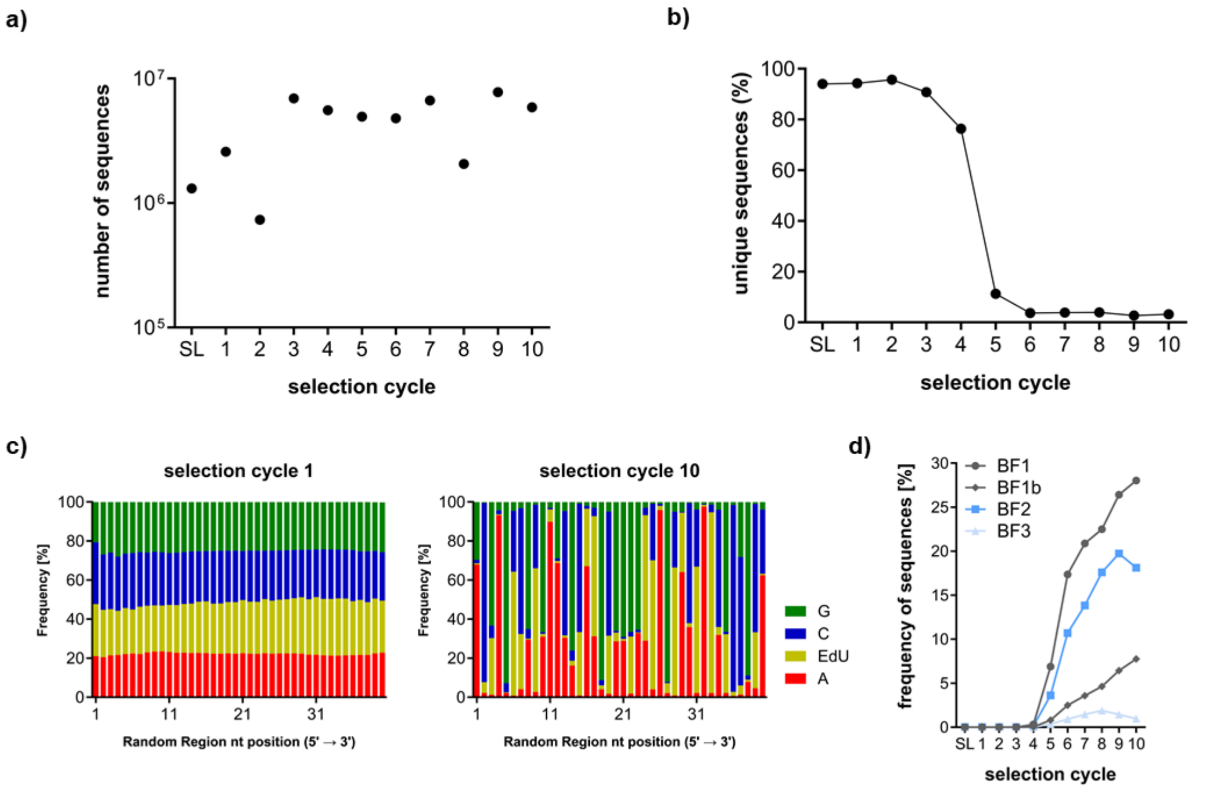

Figure S2: NGS analysis of all selection cycles of the split-combine click-SELEX A targeting CoV2-S. (**a**) Number of analysed sequences per selection cycle by NGS. (**b**) Percentage of unique sequences in the DNA libraries from selection cycles 1-10 and the starting library (SL). (**c**) Nucleotide distribution of the 42-nucleotide random region at the different positions in the selection cycles 1 and 10. (**d**) Frequency of the representative sequences BF1, BF1b, BF2 and BF3 throughout the DNA libraries from selection cycles 1-10 and the starting library (SL). The selected sequences represent the most enriched sequences in the selection. BF1b is a point mutant of sequence BF1.

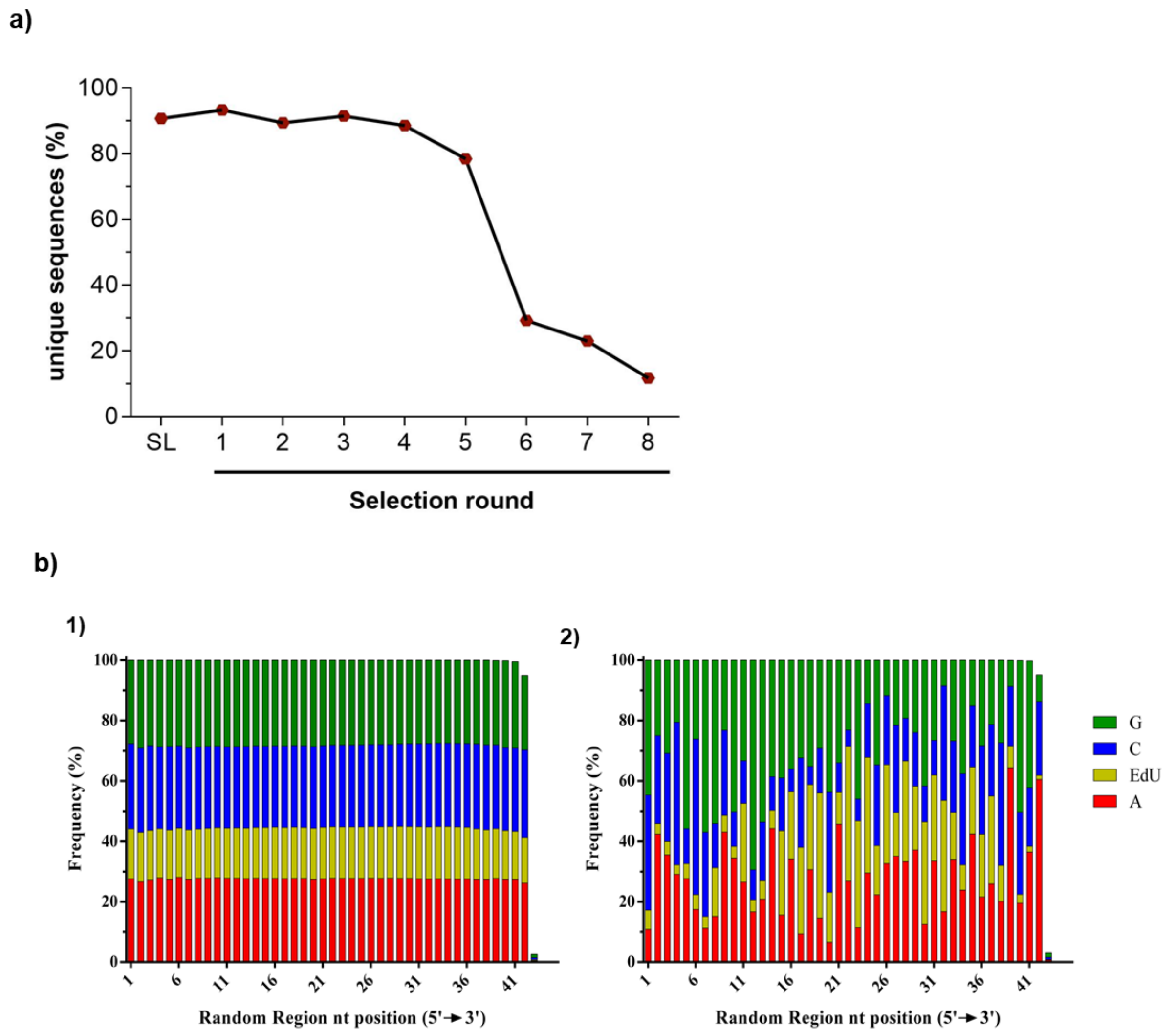

Figure S3: NGS analysis of all selection cycles of the split-combine click-SELEX B targeting CoV2-S. (a) The percentage of unique sequences for each selection round population and starting library (SL). (**b)** The statistical nucleotide distribution of the random region of 1) SL and 2) R8 library. a uniform distribution of the four nucleobases is revealed by NGS and the mean abundance of the EdU residues in the library is approximately 16.8%, resulting in an average of 7 EdU nucleotides per DNA strand.

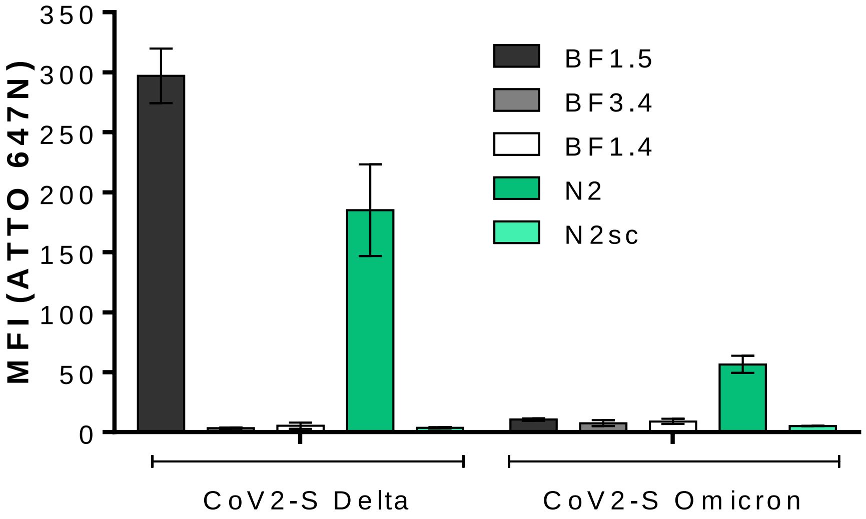

Figure S4: Interaction analysis of BF and N2 clickmers to CoV2-S Omicron variant. Flow cytometry-based interaction analysis with CoV2-S Delta and Omicron variant immobilized on magnetic beads. ATTO647N labelled, benzofuran click-modified sequences BF1.5, BF3.4 and non-binding control BF1.4 (200 nM) were incubated at 25°C in saliva-like buffer. ATTO647N labelled indole click-modified sequence N2 and non-binding control N2sc (200 nM) were incubated at 37°C in PBS buffer. (n = 2, mean +/- SD).

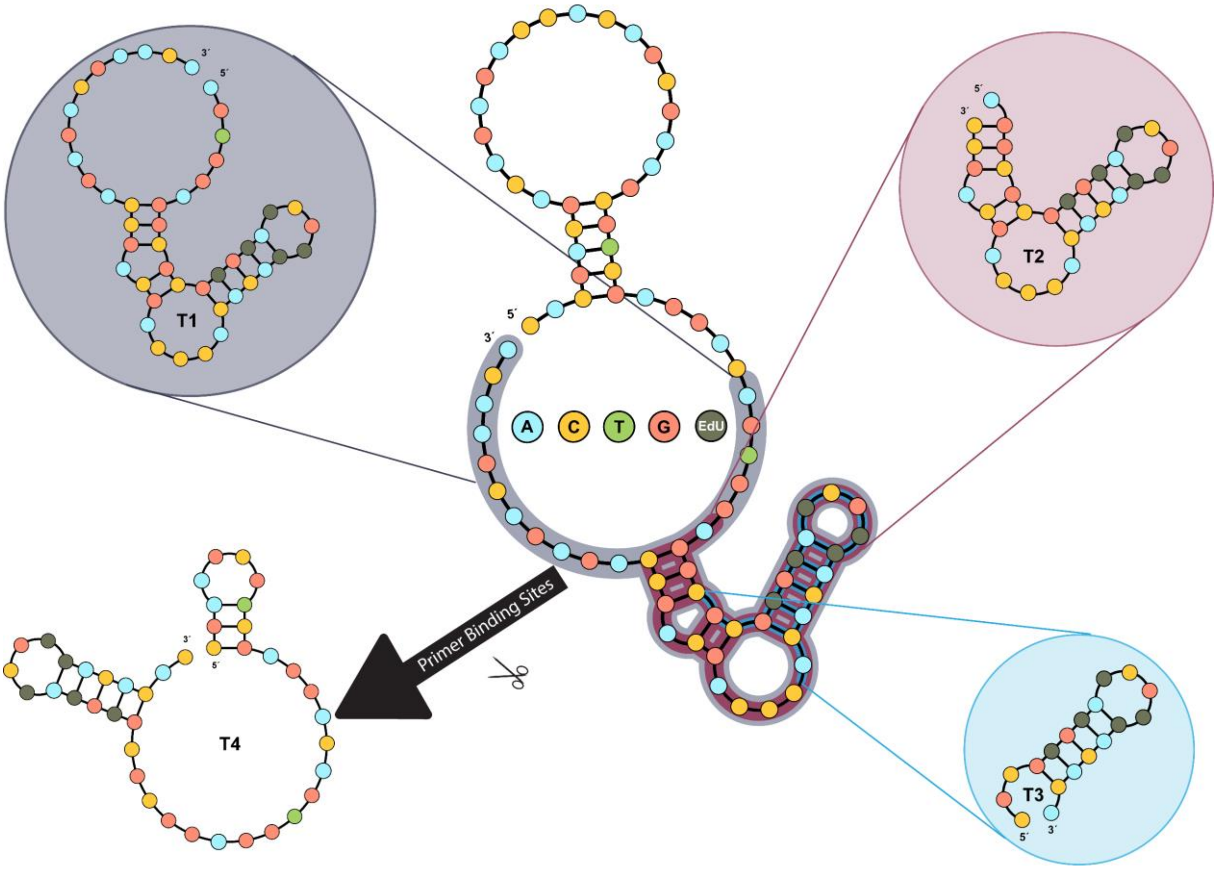

Figure S5: Secondary structure predictions of N2.5 truncates by NUPACK. Each truncated sequence was analyzed for secondary structure as an unmodified DNA oligonucleotide with EdUs replaced by dTs. All five crucial EdU residues from N2 clickmer are shown in grey for N2.5 and all four truncates. T1: 47 nt, T2: 31 nt, T3: 18 nt, T4: 42 nt.

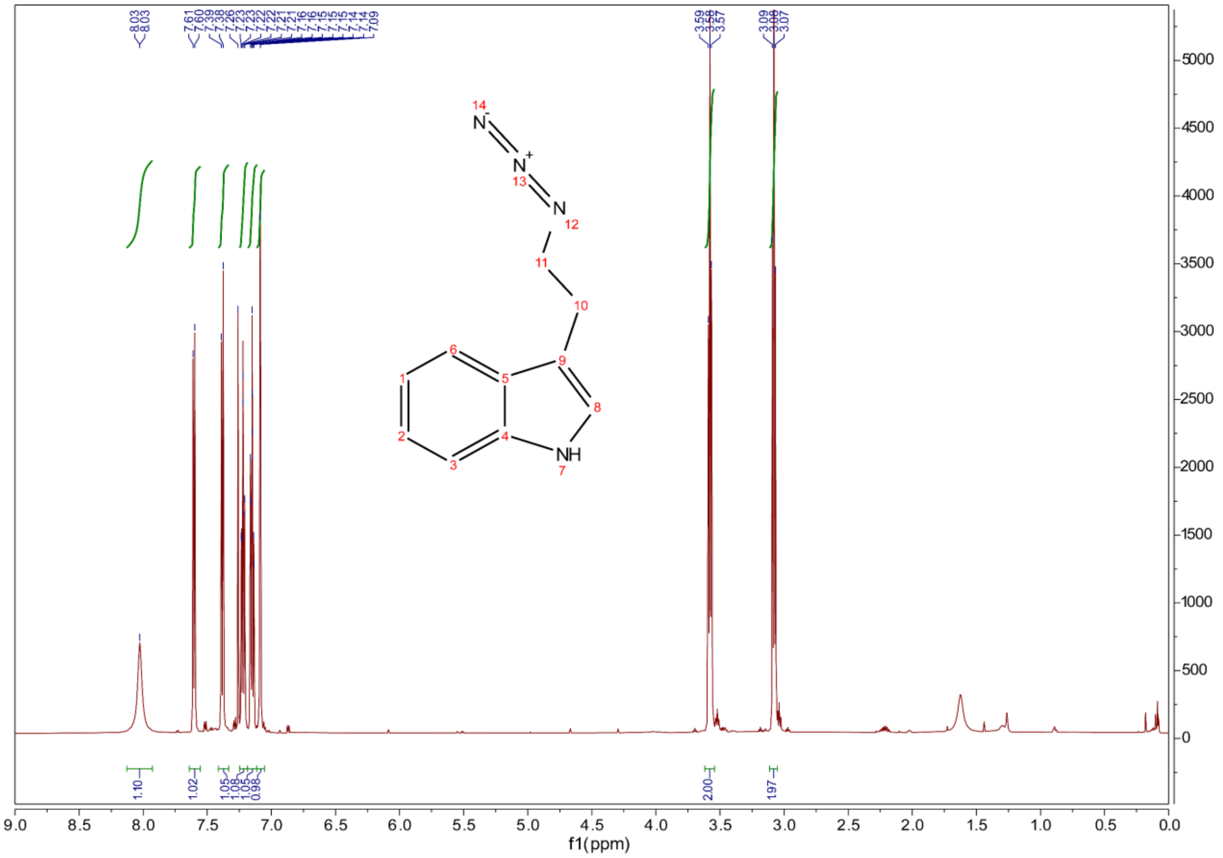

Figure S6: ^{1}H NMR spectrum of the resynthesized 3-(2-Azidoethyl)-1H-indole (300 MHz, $CDCl_3$). δ 8.03 (br s, 1H, NH), 7.61 (d, J = 2 Hz, 1H), 7.39 (d, J = 2 Hz, 1H), 7.22 (t, J = 2 Hz, 1H), 7.15 (t, J = 2 Hz, 1H), 7.09 (s, 1H), 3.58 (t, J = 2 Hz, 2H), 3.08 (t, J = 2 Hz, 2H).
